# From structure to therapy: the critical influence of cartilaginous endplates and microvascular network on intervertebral disc degeneration

**DOI:** 10.3389/fbioe.2024.1489420

**Published:** 2024-10-28

**Authors:** Yu Sun, Zhaoyong Li, Jiahao Duan, Enxu Liu, Lei Yang, Fei Sun, Long Chen, Shaofeng Yang

**Affiliations:** ^1^ Graduate School of Hunan University of Chinese Medicine, Changsha, China; ^2^ Department of Orthopaedics, The First Affiliated Hospital of Hunan University of Chinese Medicine, Changsha, China

**Keywords:** cartilage endplate, microvessels, intervertebral disc degeneration, Modic changes, nutrient diffusion

## Abstract

The intervertebral disc (IVD) is the largest avascular structure in the human body. The cartilaginous endplate (CEP) is a layer of translucent cartilage located at the upper and lower edges of the vertebral bodies. On one hand, CEPs endure pressure from within the IVD and the tensile and shear forces of the annulus fibrosus, promoting uniform distribution of compressive loads on the vertebral bodies. On the other hand, microvascular diffusion channels within the CEP serve as the primary routes for nutrient supply to the IVD and the transport of metabolic waste. Degenerated CEP, characterized by increased stiffness, decreased permeability, and reduced water content, impairs substance transport and mechanical response within the IVD, ultimately leading to intervertebral disc degeneration (IDD). Insufficient nutrition of the IVD has long been considered the initiating factor of IDD, with CEP degeneration regarded as an early contributing factor. Additionally, CEP degeneration is frequently accompanied by Modic changes, which are common manifestations in the progression of IDD. Therefore, this paper comprehensively reviews the structure and physiological functions of CEP and its role in the cascade of IDD, exploring the intrinsic relationship between CEP degeneration and Modic changes from various perspectives. Furthermore, we summarize recent potential therapeutic approaches targeting CEP to delay IDD, offering new insights into the pathological mechanisms and regenerative repair strategies for IDD.

## 1 Introduction

Low back pain (LBP) has long been recognized as one of the primary factors affecting human quality of life. With the gradual intensification of population aging, the substantial socio-economic burden caused by LBP continues to accumulate (Genedy et al., 2024; Abel et al., 2024). Intervertebral disc degeneration (IDD) is a complex pathological process involving multiple factors, including genetics, obesity, age, and lifestyle. It is primarily characterized by the loss of water content in the disc tissue, reduced elasticity, decreased height, and diminished ability to respond to mechanical loads (Xu et al., 2023). IDD and its associated pathological changes, such as lumbar osteophytes, spinal instability, and lumbar disc herniation, are significant contributors to LBP. IVDs are the largest avascular structures in the human body, and nutrient supply is crucial to their performance. The microvascular plexus within CEP acts as a “bridge,” facilitating nutrient and waste exchange within IVDs (Crump et al., 2023). This review begins with the tissue structure of the CEP and its microvascular network, using their mechanism of action in IDD as a focal point to comprehensively elucidate the crucial role of the CEP in the cascade of IDD. Additionally, we summarize current treatments targeting CEP to delay IDD, providing new insights for the prevention and treatment of degenerative spine diseases within the framework of precision medicine.

## 2 Overview of CEP and its microvascular structure

IVD is an avascular structure composed of fibrocartilage that separates vertebrae, transmits loads, and provides flexibility and effective stress support for the spine. CEP consists of semi-porous thickened cancellous bone and hyaline cartilage arranged in layers (Wang et al., 2022), encompassing the upper and lower boundaries of the IVD. It prevents the IVD from bulging into adjacent vertebrae and provides crucial mechanical support for anchoring the nucleus pulposus (NP) and annulus fibrosus (AF). Additionally, the CEP is part of the main nutritional supply network for the IVD. At the interface between the CEP and bony endplate (BEP), terminal branches of metaphyseal arteries and nutrient arteries form a dense capillary network extending into the vertebral marrow cavity, known as the “vascular network loop” or “capillary bed” (Huang et al., 2021). These vessels are primarily distributed in the central region, containing numerous hematopoietic stem cells, adipocytes, and nerves. Spinal nerves and capillaries enter the posterior cortical vertebral foramen and supply this area through small pores in the cortical shell. These “porous structures,” similar to cartilage sinuses, are abundant in the CEP, effectively increasing its permeability and thereby nourishing the NP and AF tissues (Habib et al., 2023; Ren et al., 2023). Furthermore, compared to the CEP-AF interface, the capillary network at the CEP-NP interface is denser, forming a continuous vascular bed with sinusoidal venous channels, providing critical pathways for nutrient diffusion and waste removal within the IVD.

Nutrients enter the IVD through the capillary network around the AF and nourish the disc tissue (Mohd Isa et al., 2022a; Chu et al., 2018). However, research indicates that axial diffusivity, through the CEP, is three times higher than radial diffusivity, via the AF (Travascio et al., 2009). Additionally, the electrical conductivity of the human IVD is closely related to solute transport and nutrient exchange. It has been found that the conductivity of AF tissue is directionally dependent, with significantly lower radial conductivity compared to axial conductivity under sustained mechanical stress (Jackson et al., 2009). The conclusions of these studies further underscore the critical role of the CEP in providing essential nutrients to the disc.

**FIGURE 1:**
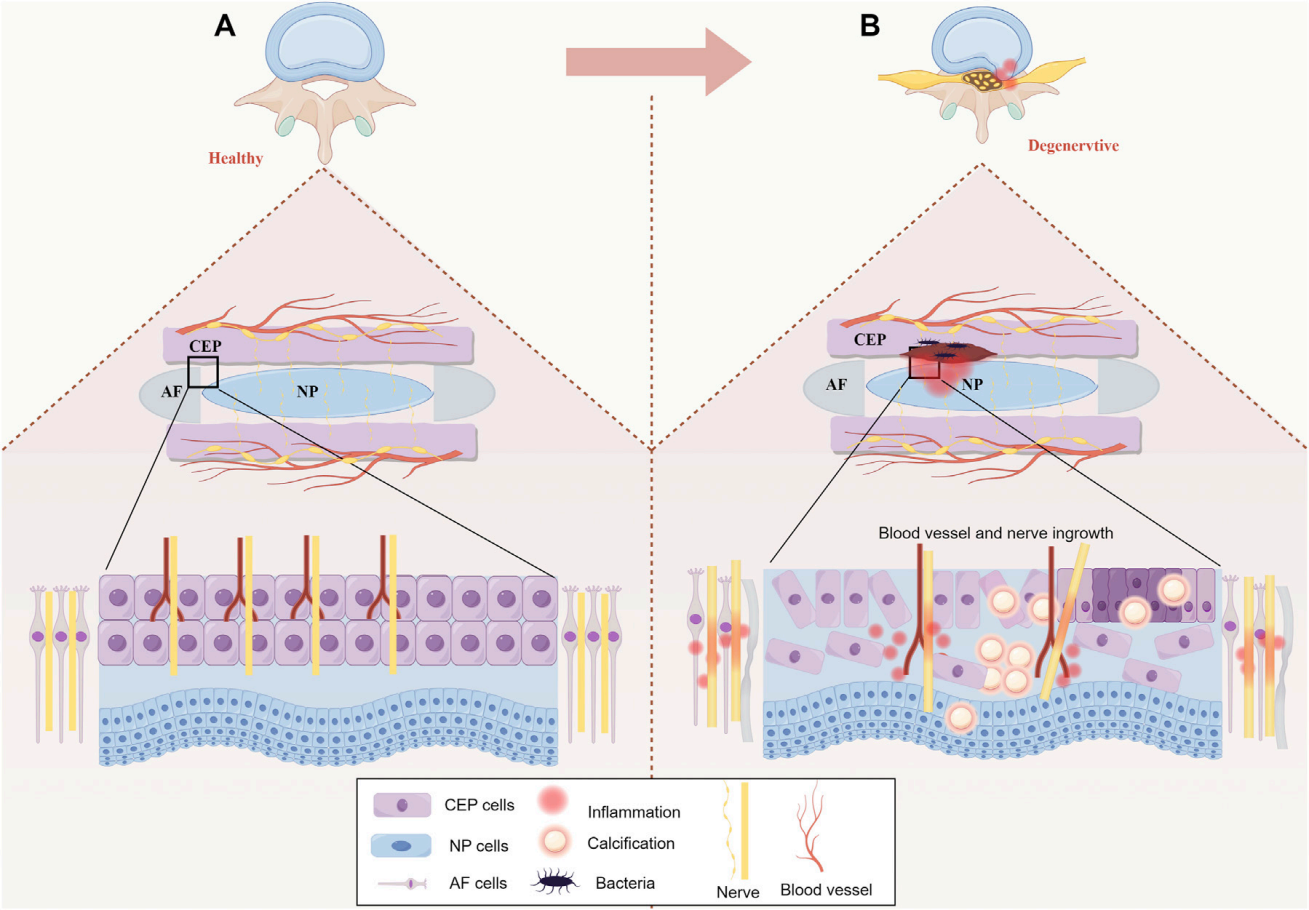
**(A)** Healthy IVD: In a healthy IVD, AF collagen fibers enter the bottom of the CEP parallel to the bone. The collagen fibers of NP partially permeate the CEP. The CEP is penetrated by vascular plexus from the vertebral bone cancellous, in which the epiphyseal artery and the trophic artery end branch formed a dense capillary network. **(B)** Degenerative IVD: The degenerative CEP has reduced thickness, calcification and fibrosis of the inner and outer layers leading to apoptosis, in addition to progressive atrophy of the vascular bed within the CEP, hardening of the adjacent bone endplate and progressive stripping from the CEP. At the same time, the binding between NP and AF becomes weaker.

Degeneration of the CEP involves a reduction in microvessels and decreased permeability, leading to an imbalance in energy metabolism within the IVD and inducing IDD. Therefore, we believe that the lack of effective nutrient supply in IVD is the prerequisite for inducing IDD, and the microstructural changes in the vascular plexus within CEP are an important cause of IDD formation. Additionally, the neural supply to the CEP is comparable to that of the IVD. Nerve endings are mainly located in the outer layer of the CEP and spread toward the central part. Similarly, in healthy IVDs, nerve endings extend to the outermost layer of the AF (Din et al., 2022; Lai et al., 2023). These nerves send nociceptive impulses to the sympathetic nervous system; thus, LBP induced by IDD is closely related to CEP degeneration. In addition, occult intervertebral disc infection has also been considered as a potential factor for intervertebral disc degeneration (Rollason et al., 2013; Fritzell et al., 2004). Endplate and its microvascular plexus injury may form a channel for bacteria to enter the intervertebral disc, while the low oxygen tension and low pH of the intervertebral disc create favorable conditions for bacterial proliferation (Albert et al., 2013). The internal structure of healthy and degenerative IVD is shown in Figure 1.

## 3 Overview of the mechanism of CEP degradation in the IDD cascade reaction

IDD is a common and complex degenerative spinal disease influenced by various factors, including natural degeneration, genetics, immunity, injury, and smoking. These factors contribute to CEP degeneration through multiple pathways, ultimately inducing IDD. The following is a review of common factors contributing to CEP degeneration.

### 3.1 Natural degeneration

In humans, the degeneration process of the CEP begins at the age of 2. As skeletal development gradually matures after adulthood, the microenvironment within the CEP is affected by various factors, accelerating cell apoptosis and gradually forming calcified foci, which are subsequently replaced by bone tissue (Ashinsky et al., 2021). The density of the microvascular network within ossified CEP decreases, reducing nutrient and metabolic product diffusion between the IVD and CEP. Additionally, during the human growth period, collagen type II (COL2) and proteoglycans, main components of the extracellular matrix (ECM), are synthesized in greater quantities, with relatively active COL2 denaturation. Upon reaching maturity, the functions of COL2 and proteoglycans significantly weaken. During the degenerative phase, COL2 denaturation is accompanied by increased COL1 synthesis. At this time, the CEP secretes collagen type X, a protein related to chondrocyte hypertrophy and calcification, which can serve as a serum marker for assessing CEP degeneration and targeted IDD therapy (Berg-Johansen et al., 2018).

### 3.2 Genetic factors

Both CEP degeneration and IDD exhibit a typical familial genetic predisposition. Clinically, several epidemiological studies have demonstrated a strong genetic predisposition to IDD. A twin study revealed that up to 74% of the variation in lumbar disc degeneration could be attributed to genetic factors (Battié et al., 1995). Additionally, a genetic study of cervical and lumbar spine degeneration in twins reported heritability estimates of 73% and 74%, respectively (Sambrook et al., 1999). These results further suggest a significant genetic influence on the variation in disc degeneration. Furthermore, another study confirmed the existence of familial predisposition to IDD, with heritability estimates ranging from 34% to 61% across different spinal regions. Segregation analysis indicates that the mode of inheritance is complex, involving multiple factors and genes that likely contribute to intergenerational transmission (Kalichman and Hunter, 2008). This genetic susceptibility is largely driven by key extracellular matrix (ECM) components, such as COL2, COL9, and COL11.

The expression of COL2, a crucial ECM component, is closely related to the occurrence and development of IDD. Sahlman et al. (2001) investigated the role of COL2A1 gene heterozygous inactivation in early IDD by knocking out the COL2A1 gene in mouse bone tissue. Their results demonstrated that the CEP in 1-month-old heterozygous knockout mice was thicker, more irregular, and showed early calcification compared to control mice. Similarly, COL9A2 plays a critical role in maintaining the normal phenotype of the CEP. A study analyzing the entire spinal system of mice after knocking out the COL9A2 gene found that the loss of the COL9A2 gene led to CEP remodeling in IVD tissues, inhibited ECM synthesis, and accelerated matrix degradation and chondrocyte hypertrophy (Kimura et al., 1996). Furthermore, a mutation in the human COL9A2 chain coding gene causes one of the glutamine codons to become tryptophan, significantly increasing the probability of disc degeneration in patients with the mutated gene (Karppinen et al., 2002). In addition, Stickler syndrome caused by mutations in the COL11 coding gene is also often accompanied by CEP calcification and IDD (Soh et al., 2022).

### 3.3 Immune inflammatory response

As mentioned, the IVD is the largest avascular structure in the human body, with nutrient diffusion pathways within the microvascular plexus of the CEP. The dense ECM within the IVD, due to high physical pressure and high proteoglycan concentrations, hinders the growth of nerves and blood vessels in non-degenerate IVD tissue. Therefore, the CEP is considered a natural barrier between the blood and the IVD (Sun et al., 2020). It effectively isolates the AF, NP, from blood circulation and the host immune system. When the CEP is intact, the IVD is considered immune-privileged tissue lacking immune cells. Native IVD cells produce numerous cytokines and chemokines, including IL-1, TNFα, IL-6, IL-8, and IL-17 (Bermudez-Lekerika et al., 2022). During CEP degeneration, these factors are upregulated, driving catabolic processes in the IVD through paracrine or autocrine mechanisms, creating conditions for anaerobic bacteria growth in degenerate IVD. These bacteria produce a large number of inflammatory factors, thereby recruiting more inflammatory cells such as T cells, B cells, dendritic cells, and macrophages, making degenerate IVD an ideal site for microbial growth and accumulation of metabolic products (Abdel Fattah and Nasr El-Din, 2021). Additionally, abnormal mechanical stress and sustained axial loading can lead to irreversible microdamage to the CEP, providing an effective pathway for the migration of numerous immune cells such as T cells, B cells, macrophages, and mast cells to the IVD (Gorth et al., 2018). Other studies have shown that native CEP cells can also perform immune cell-like functions, such as phagocytosis, effectively mitigating a series of immune-inflammatory responses triggered by apoptotic or immunogenic cells (Bermudez-Lekerika et al., 2022).

### 3.4 Degeneration after injury

When the spine experiences long-term high-load axial pressure exceeding the CEP’s maximum threshold, its weak central part gets damaged, allowing the NP to protrude through the CEP into the vertebral cancellous bone, eventually forming Schmorl’s nodes. The formation of Schmorl’s nodes is a result of chronic damage to the CEP and is an important pathological change in the process of IDD (Teraguchi et al., 2024). Considerable controversy still exists regarding the pathophysiological mechanism of Schmorl’s node formation. The classic hypothesis suggests that Schmorl’s nodes are related to abnormal changes in vertebral blood vessels during development. During the fetal period, IVD nutrition is supplied by internal capillaries, which subsequently degenerate, making normal adult IVD avascular tissues. According to this theory, abnormalities in vascular channels weaken CEP function, promoting NP protrusion into the subchondral bone (Su et al., 2023; Zhao et al., 2010). Autopsies of adult lumbar spines have found that these microvascular channels penetrating the CEP are mainly distributed in the central area of the vertebral surface, which is also the most common site of Schmorl’s nodes (Nachemson et al., 1970). Additionally, other scholars have conducted full-thickness histological examinations of Schmorl’s nodes and found extensive fibrosis under the CEP and within the marrow cavity, with a significant reduction in adipocytes and osteocytes within the trabeculae (Peng et al., 2003). This indicates that ischemic necrosis under the CEP may be the main cause of Schmorl’s nodes, and NP herniation into the vertebrae is a secondary phenomenon of this pathological process, similar to the pathophysiological process of avascular necrosis of the femoral head. Further studies suggest that Schmorl’s nodes may develop from tiny cracks in the CEP. These small injuries are extremely difficult to capture through imaging during clinical diagnosis and are often discovered during biopsy (Pfirrmann and Resnick, 2001).

### 3.5 Smoking

**FIGURE 2:**
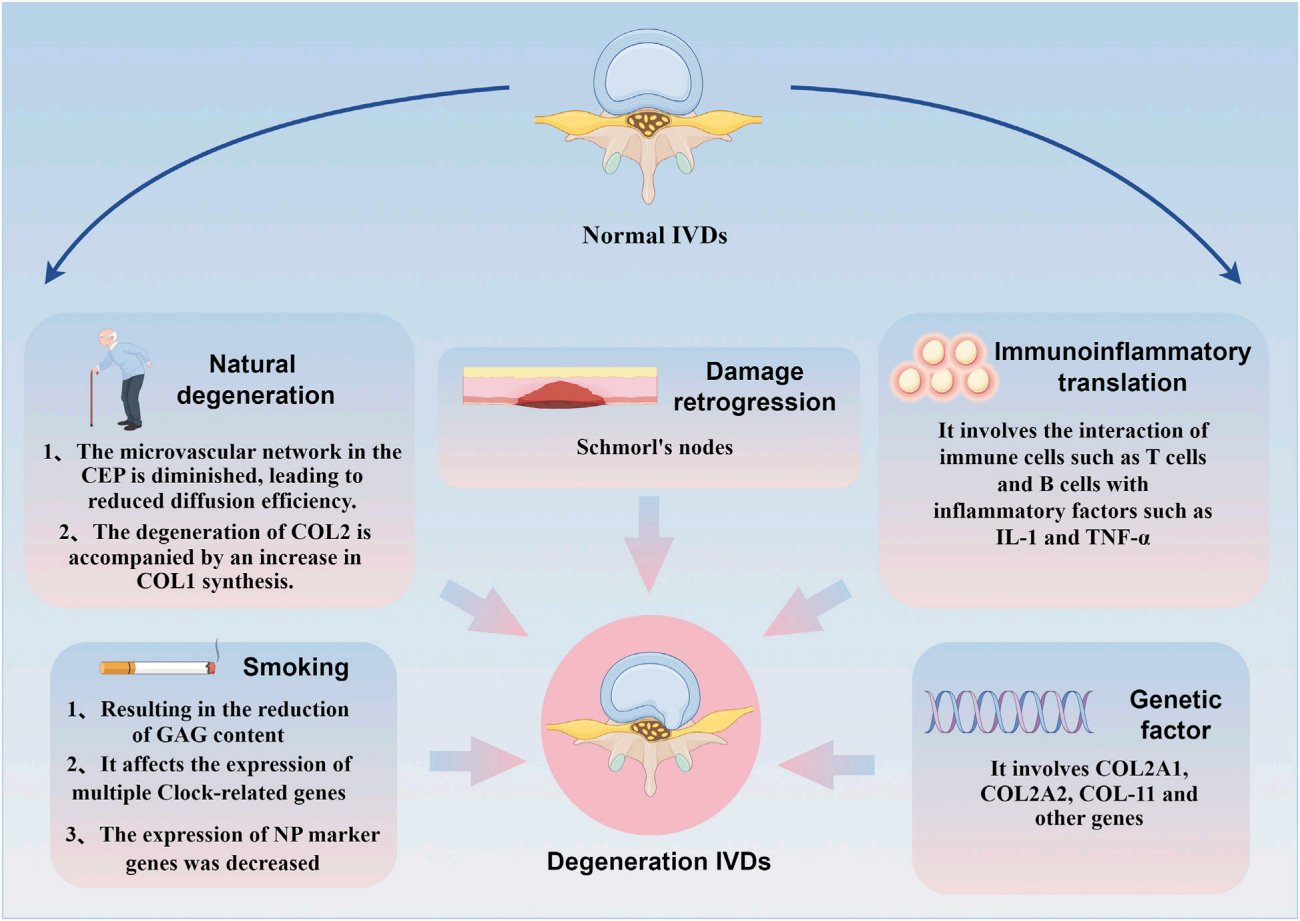
Brief mechanism of CEP degradation mediated by different factors in the IDD cascade reaction. Natural degeneration, genetics, immunity, injury and smoking can affect CEP degeneration through multiple pathways and induce IDD.

Smoking is another risk factor that induces CEP degeneration and accelerates IDD. Glycosaminoglycan (GAG) is a class of functionally rich linear acidic polysaccharides that bind to core proteins through serine residues in the form of covalent bonds to form proteoglycans, mainly present in the ECM (Silagi et al., 2018). Studies have found that the reduction of GAG content is the most significant biochemical change in IDD. The loss of GAG directly impairs the mechanical function of IVD in the spinal system (Huang et al., 2023). Nicotine intake significantly reduces the GAG concentration around the CEP while inducing contraction of the microvascular network within it, reducing the exchange of nutrients and anabolic substances between blood vessels (Elmasry et al., 2015). The impact of passive smoking on IDD should not be ignored. Studies have shown that although second-hand smoke has a weaker effect on the gene expression profiles of AF, CEP, and NP, it significantly affects the expression of several clock-related genes. These genes are expressed in a circadian rhythm in IVD cells, with most showing a 6–9 h phase shift when induced by passive smoking (Numaguchi et al., 2016). Additionally, second-hand smoke alters the expression of proteases and protease inhibitors, which reduces the expression of NP marker genes (Ogawa et al., 2005). Epidemiological studies indicate that when risk factors such as systemic disease, heavy working conditions, obesity, trauma and family history were excluded, smoking increased lumbar disc degeneration, suggesting a strong correlation between smoking and the progression of IDD (Kiraz and Demir, 2020; Chen et al., 2018). This highlights the importance of smoking cessation in preventing CEP degeneration and potentially slowing down the IDD process. Figure 2 outlines the brief mechanism of CEP degeneration in the cascade reaction of IDD, see Figure 2.

## 4 Discussion on the correlation between CEP degeneration and Modic nodules

Changes in MRI signal intensity of vertebral bone marrow around the IVD due to CEP injury are referred to as Modic changes. Based on T1 and T2-weighted MRI, Modic changes are classified into three types. Modic Type I: low signal on T1-weighted images, high signal on T2-weighted images, histologically characterized by CEP edema, immune cell infiltration, fibrogenic stromal cell proliferation, and fibrous vascular granulation tissue expansion. Modic Type II: high signal on T1-weighted images, mildly high or equal signal on T2-weighted images, characterized by CEP fissures or microfractures. Modic Type III: low signal on both T1 and T2-weighted images, indicating sclerotic changes in CEP tissue (Wang et al., 2023). Since CEP injury often accompanies Modic changes and is a key factor in inducing IDD, it may serve as the bridge in studying the relationship between Modic changes and IDD. In-depth exploration of the mechanisms between CEP and Modic changes is clinically significant for delaying IDD. The following review explores the relationships between Modic changes, CEP injury, and IDD from different perspectives.

On one hand, CEP injury is accompanied by Modic changes. Due to CEP injury, the barrier function between CEP and BEP is compromised, weakening its role as a filter for cells and macromolecules, leading to interactions between the degenerated IVD and adjacent vertebrae. Matrix metalloproteinases (MMPs) and other proteins escape into adjacent bone marrow through damaged CEP channels, triggering ECM degradation. ECM degradation products and inflammatory mediators within the IVD, even without infiltrating it, further exacerbate Modic changes, advancing the progression of IDD (Liu et al., 2019; Feng et al., 2021). Therefore, CEP injury accompanied by Modic changes promotes inflammatory crosstalk between the IVD and vertebrae, leading to widespread degeneration in vertebral segments with Modic changes. Additionally, increased concentrations of cytokines and chemokines produced during IVD degeneration can more easily diffuse from the IVD into adjacent vertebrae, triggering immune cell activation.

**FIGURE 3:**
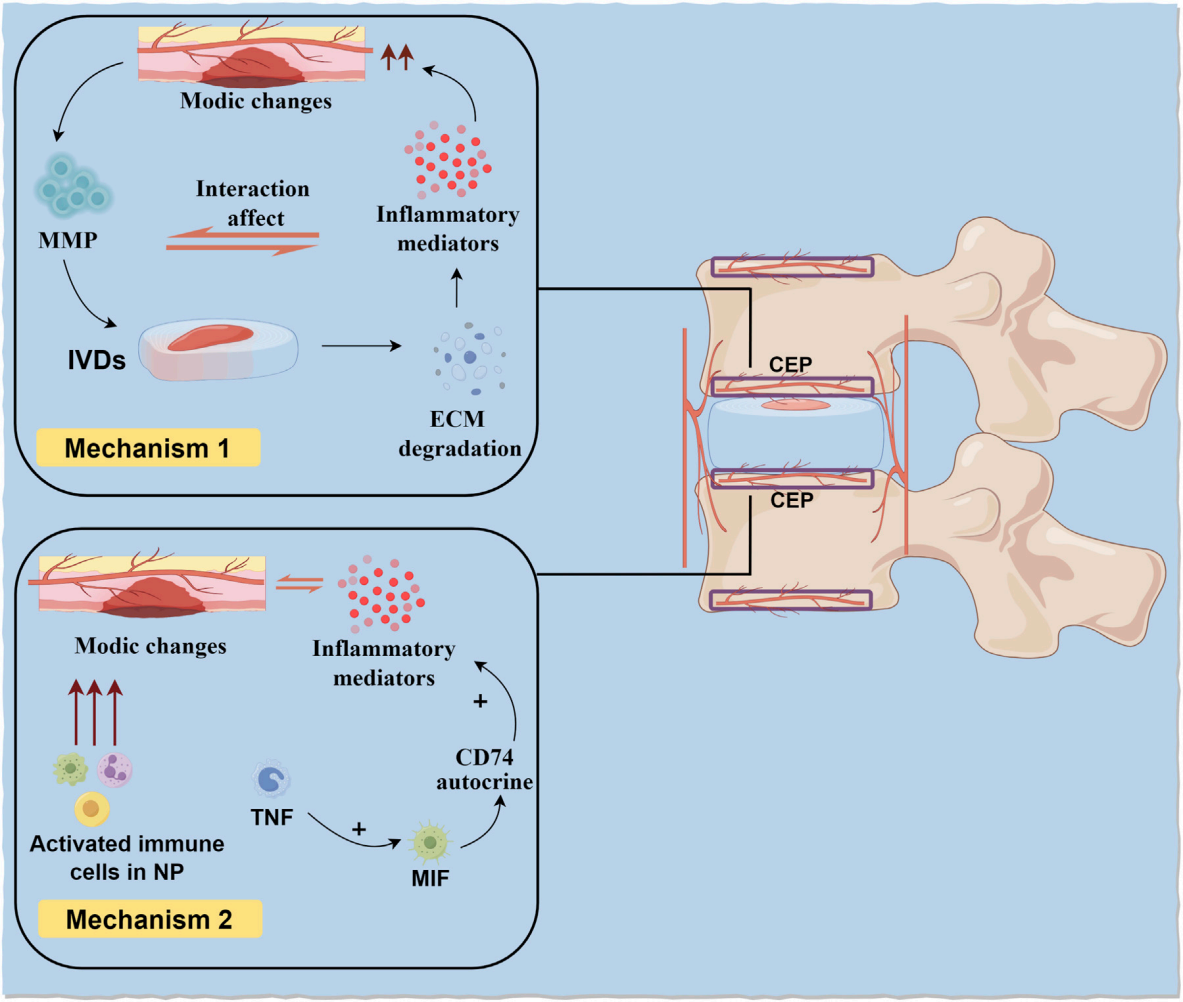
CEP damage and Modic changes. On the one hand, CEP damage accompanied by Modic changes promotes inflammatory crosstalk between IVD and vertebral body. On the other hand, Modic changes cause substances in NP to directly contact with the body’s immune system and produce immune rejection, leading to damage to adjacent CEP structures.

On the other hand, Modic changes exacerbate CEP injury. As previously mentioned, due to immune isolation during embryonic development, substances within the NP have immune privilege with the body’s immune system. Modic changes cause substances within the NP to directly contact the immune system, triggering an immune rejection response and damaging adjacent CEP structures (Heggli et al., 2024). Further research indicates that Modic changes can induce fibrosis and pro-inflammatory responses between the NP and adjacent IVD (Dudli et al., 2023). Additionally, other studies have found that activated immune cells in the NP after Modic changes may cause CEP damage (Heggli et al., 2022). At the cellular level, after Modic changes, CEP cells express more tumor necrosis factor (TNF), macrophage migration inhibitory factor (MIF), and its receptor CD74. Specifically, TNF upregulates MIF in CEP cells, and MIF exacerbates the secretion of pro-inflammatory factors in CEP cells through an autocrine mechanism involving CD74. This positive inflammatory feedback loop suggests that the CEP can independently exacerbate the inflammatory response in Modic changes, separate from the IVD (Xiong et al., 2014; Ohtori et al., 2006). The interaction mechanism between Modic changes and CEP injury is shown in Figure 3.

## 5 Modern medical research targeting CEP to delay IDD

The most common treatments for degenerative spine diseases currently include surgical and non-surgical methods. However, neither treatment fully alleviates complications caused by IDD, such as LBP, nor do they restore the biomechanical function of the IVD. Regenerative medicine and tissue engineering are current hotspots in the research field of degenerative spine diseases, capable of targeting specific tissues according to the different stages of IDD evolution. In the early stages of IDD, cellular activity within the IVD tissue is relatively good and still responds to external stimuli to maintain ECM balance. At this stage, molecular therapy is the better option to delay IDD. In the mid-stages of IDD, the activity and number of endogenous stem cells within the IVD tissue decline. At this time, cell transplantation and stem cell-rich plasma infiltration therapy are advisable. In the late stages of IDD, there is a severe imbalance of cells and ECM within the IVD tissue. At this stage, cell scaffolds and biomaterials guided by tissue engineering should be the primary choice. The following is a systematic review of recent advanced therapeutic approaches targeting the CEP to delay IDD progression, providing new insights into the pathological mechanisms and regenerative repair strategies for IDD.

### 5.1 Molecular therapy

In the early stages of IDD, nutrient supply is critical for the anabolic metabolism of IVD cells. Molecular therapy, as a novel treatment, involves injecting growth factors or other molecules into the IVD to stimulate cell growth, increase ECM anabolic metabolism, and restore physiological balance within the IVD (Vadalà et al., 2015). Molecular therapy targeting the CEP usually focuses on increasing IVD tissue permeability. Researchers found that injecting calcium-binding compounds (novel recombinant protein copolymers) into the CEP for thorough decalcification significantly improved nutrient supply to the IVD (Boyd and Carter, 2006). Treating degenerated CEP tissue with trypsin or hyaluronidase to remove large proteoglycans also delays its degeneration. Matrix metalloproteinase 8 (MMP8) is a zinc-dependent endopeptidase with high selectivity for aggrecan and COL2, and it degrades various protein components of the ECM. Studies have shown that injecting MMP8 into the CEP, linked with large nanoparticles that cannot migrate to other tissues, maximally avoids NP or AF degeneration caused by off-target digestion or matrix fragments, ultimately promoting solute absorption and nutrient diffusion within the CEP, effectively delaying IDD (Dolor et al., 2019). However, it is important to assess the potential long-term risks associated with molecular therapy. Other studies suggest that molecular therapy may accelerate CEP injury-induced IDD, possibly related to the disruption of energy supply and demand within the IVD (Wu et al., 2013).

While molecular therapy holds promise, potential long-term complications must be considered. For example, repeated injections may lead to tissue irritation or chronic inflammation, potentially exacerbating degenerative changes rather than ameliorating them (Kennon et al., 2018). Furthermore, there is concern regarding the long-term effects of growth factor therapy, which may inadvertently stimulate excessive cellular proliferation, leading to cellular mutations or unregulated ECM production. This unregulated response could predispose patients to cartilage degeneration or contribute to a recurrence of IDD symptoms over time (Sakai and Grad, 2015). Therefore, a careful evaluation of these risks is essential to maximize the benefits of molecular therapy while minimizing potential adverse effects.

As the bridge of IVD energy metabolism, the state of the CEP—whether healthy, thinned, or calcified—directly interferes with its internal supply and demand balance. Therefore, researchers suggest that when applying molecular therapy, the poor nutritional environment within the IVD, especially the degenerative status of the CEP, must be considered to achieve maximum benefit while minimizing long-term complications (Hassan et al., 2021).

### 5.2 Cell transplantation

Cell transplantation is currently a mature and widely recognized treatment method in the research of delaying IDD. Both autologous and allogeneic cells have been used in clinical trials. Autologous cells are the ideal cell source, as they do not transmit diseases and have no immune rejection. They are mostly derived from bone marrow and adipose tissue. Allogeneic stem cells have better self-renewal capabilities and face fewer ethical issues compared to embryonic stem cells (Krut et al., 2021). However, cell therapy still faces many challenges. On one hand, transplanted cells need to survive in the harsh IVD environment of hypoxia, acidity, and inflammatory factors. On the other hand, to ensure the survival rate of transplanted cells, they need to secrete sufficient nutrients to stimulate stem cell differentiation and promote ECM synthesis (Hu et al., 2023; Richardson et al., 2016). Moreover, while autologous cell transplantation generally poses a lower risk of immune rejection, challenges remain concerning the longevity and efficacy of transplanted cells. Potential complications include incomplete integration with host tissue, leading to recurrence of degeneration (Trounson and McDonald, 2015). Allogeneic cells, although beneficial in terms of proliferation, carry risks of immune rejection and potential tumorigenesis, necessitating immunosuppressive therapies that can have their own adverse effects (Martin et al., 2024; Caldwell et al., 2021). Therefore, It is essential to evaluate the long-term implications of cell transplantation. Concerns regarding immune rejection, especially with allogeneic cells, should be addressed, as well as the possibility of cellular mutations or oncogenic transformations over time. These factors could significantly impact the overall efficacy and safety of cell transplantation therapies.

Liang et al. (2017) successfully isolated human degenerated CEP cells using an agarose suspension culture system and screened for proliferative cell clusters. They found that cells isolated from the CEP were positive for stem cell markers OCT-4, NANOG, and SOX-2, as well as common BM-MSC markers CD105, CD73, CD90, and Stro-1. These results were the first to confirm the presence of stem cells in human degenerated CEP. Additionally, the research team found that using CEP stem cells isolated from healthy individuals to stimulate NP cells resulted in exosomes released by NP cells activating the PI3K/AKT signaling pathway, thereby reducing apoptosis rates (Luo et al., 2021). Therefore, exosomes derived from CEP stem cells may be an effective way to delay IDD. However, current research achievements in cell therapy targeting the CEP to delay IDD are limited.

### 5.3 Tissue engineering and regenerative medicine treatment

Besides the aforementioned treatments, recent advancements in tissue engineering and regenerative medicine have provided new possibilities for delaying IDD. Compared to molecular therapy and cell transplantation, the primary distinction of tissue engineering is its focus on the reconstruction and regeneration of the IVD. Composite cell biomaterials are the main tools for reconstructing the structure and function of degenerated IVDs. In theory, biological scaffolds can provide a three-dimensional space for cells to maintain morphological and functional stability, making regenerative repair more effective (Mohd Isa et al., 2022b). In tissue engineering, biological scaffolds need to withstand biomechanical stress from the spine and provide a suitable growth environment for stem cells, enabling them to stimulate cell growth and promote ECM anabolic metabolism *in vivo* (Jarrah et al., 2023).

However, the long-term integration of these engineered tissues into the host remains a challenge. Scaffolds that do not adequately integrate can lead to chronic inflammatory responses, causing further degeneration of the IVD (Che et al., 2022). Additionally, the risk of mechanical failure under physiological loads raises concerns regarding their durability and effectiveness over time (Lin and Kang, 2021). The potential for scaffold materials to degrade and release particles into the surrounding tissues could also pose a risk of adverse reactions, such as inflammation or even osteolysis (Maherani et al., 2024; Pereira et al., 2020). Therefore, obtaining materials with mechanical properties comparable to those of natural IVDs has been a major bottleneck in the field. In addition, continuous evaluation of the mechanical and biological properties of these scaffolds *in vivo* is essential to ensure their safety and efficacy for long-term use.

Studies have shown that co-culturing scaffolds seeded with AF or NP cells and scaffolds seeded with chondrocytes can produce native interface characteristics (Chong et al., 2020; Arana et al., 2010). However, even if COLⅠ, COLⅡ, and aggrecan are similarly distributed in IVD tissue as in native tissue, their mechanical strength is weaker than native tissue, meaning they cannot perform their expected biomechanical functions under daily mechanical loads (Chong et al., 2020). Therefore, other studies have explored CEP-modified IVD-like annular structures (eDAPS) in rat and goat models as ideal materials for IVD replacement. In this structure, the CEP is composed of porous polyethylene caprolactone (PCL) foam combined with NP and AF components. Their research shows that after 20 weeks of fixation, native cells from adjacent tissues migrate into the CEP structure, gradually producing matrix components and sparse blood vessels. This process is crucial for reversing CEP degeneration and promoting nutrient diffusion in the IVD (Gullbrand et al., 2018). This indicates that developing functional NP and AF regenerative materials, along with promoting their stable integration with the CEP, leads to more efficient IVD reconstruction. However, it should be noted that while vascularization significantly impacts delaying IDD when targeting the CEP, if it occurs within NP tissue, it may accelerate IDD progression and exacerbate clinical symptoms such as LBP (Williams et al., 2021).

3D bioprinting is a commonly used technique in tissue engineering research, but it has many problems. In recent years, researchers have used bio-inks like alumina platelets or carbon fibers to enhance structural strength and recreate load-bearing tissues like the CEP, but the printing resolution has greatly limited progress (De Pieri et al., 2020). Additionally, using decellularized ECM is another option in this field, which not only solves issues like low printing resolution but also aids in the design of 3D printed scaffolds. However, the mechanical properties of chemically modified decellularized ECM are weaker compared to native tissue. Its poor scalability, low reproducibility, and high manufacturing costs also constrain the development of IVD constructs (Liu et al., 2023). The progress of modern medical research on targeting CEP to delay IDD is shown in Table 1.

**TABLE 1:** Modern studies targeting CEP to delay IDD.

Methods of treatment	Methods of intervention	Mechanism	References
Molecular Therapy	CEP + novel recombinant protein copolymers	To promote the full decalcification of CEP and improve the nutrition supply of IVD	Boyd and Carter (2006)
	CEP + Trypsin or Hyaluronidase	The large proteoglycans in CEP were removed to delay its degeneration	Boyd and Carter (2006)
	CEP + MMP8+large nanoparticles	It avoids the degeneration of NP or AF caused by off-target digestion or matrix fragmentation, and ultimately promotes solute absorption and nutrient diffusion in CEP	Dolor et al. (2019)
Cell transplantation	CEP stem cells	NP cells were stimulated to release exosomes, and the PI3K/AKT signaling pathway was activated to reduce the apoptosis rate	Luo et al. (2021)
Tissue engineering and regenerative medicine	CEP-modified IVD-like annular structures (eDAPS)	Promote the targeted migration of native cells from adjacent tissues into the CEP structure and generate matrix components and sparse blood vessels	Gullbrand et al. (2018)

## 6 Research on traditional Chinese medicine targeting cep to delay IDD

For millennia, traditional Chinese medicine has been employed in Asian countries such as China, Japan, and South Korea to treat various orthopedic diseases, including IDD. Curcumin, the primary active ingredient in the traditional Chinese medicine turmeric, exhibits anti-inflammatory, antioxidant, and cell differentiation-inducing properties. Researchers created a cervical CEP cell degeneration model using intermittent cyclic tension stimulation and applied curcumin intervention to monitor changes in CEP cell function and metabolism under high-intensity tension load. The findings revealed that curcumin can suppress apoptosis via the autophagy pathway, decrease phenotype loss of CEP cells induced by high-intensity tension, and mitigate IDD triggered by mechanical imbalance (Xiao et al., 2020). Salvianolic acid A, an important active component of the traditional Chinese medicine Salvia miltiorrhiza, effectively improves tissue vascular permeability. Studies have shown that Salvianolic acid A can target miR-940 and miR-576-5p to alleviate IL-1β-induced degradation of the extracellular matrix in CEP cells (Zhan et al., 2023). Amygdalin is the main pharmacological component of the traditional Chinese medicine almond. Research has found that amygdalin targets the NF-κB signaling pathway, inhibits TNF-α and MMP-13, increases Col-2 expression, and reduces CEP calcification (Jiao et al., 2023; Zeng et al., 2023). Similarly, chlorogenic acid is the main antibacterial and antiviral pharmacologically active component of honeysuckle. Studies have shown that chlorogenic acid can delay the progression of IDD by inhibiting NF-κB signaling in the CEP (Ge et al., 2021). Icariin, a flavonoid compound extracted from the traditional Chinese medicine Epimedium, possesses multiple biological activities, including anti-inflammatory, antioxidant, anti-apoptotic, and anti-cancer properties (El-Shitany and Eid, 2019). Studies have shown that icariin protects against CEP degeneration and calcification under inflammatory stimulation. This protective mechanism is likely associated with the activation of mitochondrial autophagy and the inhibition of ferroptosis mediated by Nrf-2/HO-1 (Shao et al., 2022).

Traditional Chinese medicine (TCM) formulas, evolved based on TCM theoretical systems, adhere to the core concepts of holistic view and syndrome differentiation and treatment. When used to delay IDD, they achieve better synergistic therapeutic effects. Research has shown that the Shen Sui Tong Zhi formula can target the NF-κB signaling pathway in CEP cells, modulate the expression of the key gene RELA and the phosphorylation of the key protein P65, reduce the expression of inflammatory factors and catenin proteins in the LSI surgery-induced CEP degeneration model, and upregulate the expression of antenin proteins, thus delaying IDD progression (Wang et al., 2024). Another study found that the Bushen Huoxue formula combined with EPC transplantation enhanced endothelial cell viability and tube formation ability, stimulated EPC microcirculation, and played an important role in EPC angiogenesis and delaying IDD (Xie et al., 2024). Despite recent achievements in targeting CEP with single traditional Chinese medicine (TCM) components and compound formulas to delay IDD, several shortcomings remain in this research direction. Firstly, some single TCM components exhibit strong toxicological effects. The balance and avoidance of drug toxicity and efficacy relationships need further investigation. Secondly, current research is mostly based on *in vitro* cell experiments. The metabolic regulation mechanisms of single TCM components *in vivo* and their effects on CEP still require *in vivo* experiments for verification. Lastly, research on TCM compound formulas mostly focuses on effects on a single signaling pathway, while CEP cell degeneration is caused by multiple factors. The potential synergistic effects between different signaling pathways and the specific mechanisms of TCM compound formula interventions remain to be further explored. The progress of TCM targeting CEP to delay IDD is shown in Table 2.

**TABLE 2:** Traditional Chinese medicine targeting CEP delays the progression of IDD.

	Chinese medicine monomer/compound	Source	Target	Mechanism	References
Chinese medicine monomer	Curcumin	Turmeric	Autophagy	Inhibit cell apoptosis through the autophagy pathway, reduce the phenotypic loss of CEP cells induced by high-intensity tension, and alleviate IDD caused by mechanical imbalance	Xiao et al. (2020)
	Salvianolic acid A	Salvia miltiorrhiza	miR-940、miR-576-5p	Targeting miR-940 and miR-576-5p alleviates IL-1β-induced extracellular matrix degradation in CEP cells	Zhan et al. (2023)
	Amygdalin	Almond	NF-κB	Targeting the NF-κB signaling pathway, inhibiting TNF-α and MMP-13 while increasing Col-2 expression, alleviating cartilage endplate calcification	(Jiao et al., 2023) (Zeng et al., 2023)
	Chlorogenic acid	Honeysuckle	NF-κB	Inhibition of NF-κB signaling in CEPs delays IDD progression	Ge et al. (2021)
	Icariin	Epimedium	Nrf-2/HO-1	Modulates Nrf-2/HO-1-mediated activation of mitophagy and ferroptosis, inhibiting CEP degeneration and calcification under inflammatory stimulation	Shao et al. (2022)
Chinese herbal medicine	ShenSui TongZhi formula		NF-κB	Targeting the key gene RELA and key protein P65 in the NF-κB signaling pathway to reduce the expression of inflammatory factors in CEP cells	Wang et al. (2024)
	Bushen Huoxue formula		EPCs、VECs	Enhance endothelial cell viability and tube-forming ability, accelerate CEP microcirculation	Xie et al. (2024)

## 7 Discussion

It is now a consensus that degeneration in the CEP and its microvascular network hastens IDD. This review comprehensively and systematically explains the significant role of the CEP in the cascade reaction of IDD. We found that CEP-mediated development of IDD is a slow and complex process, closely related to factors such as natural degeneration, genetics, immunity, injury, and smoking. Modic changes are the most common imaging changes in IDD, and the CEP, acting as a bridge, mediates this process through two mechanisms. In addition, this article summarizes for the first time the therapeutic modalities targeting CEP to delay the progression of IDD in recent years.

Modern therapeutic strategies targeting the CEP offer new ways to delay IDD by focusing on different stages of degeneration. Molecular Therapy is effective in the early stages of IDD, aiming to enhance nutrient supply through CEP decalcification or proteoglycan removal. Methods like injecting MMP8 improve solute absorption while avoiding damage to other tissues. However, repeated treatments may cause inflammation, disrupt energy balance, or lead to overproliferation, potentially worsening degeneration. Cell Transplantation works best for mid-stage IDD. Autologous cells reduce immune rejection risks, but their survival is limited in the harsh IVD environment. Allogeneic stem cells offer better regeneration potential but come with risks of immune rejection and tumorigenesis. Emerging research highlights the potential of CEP-derived stem cells to reduce apoptosis and promote ECM synthesis through exosome signaling, though more clinical studies are needed. Tissue Engineering and Regenerative Medicine target late-stage IDD by reconstructing degenerated IVDs using biomaterials and scaffolds. While scaffolds support cell growth and ECM production, achieving native-like strength and stability remains a challenge. Poor integration can trigger inflammation or mechanical failure. New materials like 3D-printed constructs and decellularized ECM show promise, but scalability and high production costs limit their application. Additionally, vascularization is crucial for CEP repair, though excessive growth in NP tissues may accelerate degeneration.

TCM interventions—such as herbal formulations—are highlighted as promising complementary treatments to inhibit CEP degradation. These insights bridge traditional remedies with modern scientific advancements, laying the foundation for novel treatment strategies. However, it is essential to note that many of the drugs referenced for IDD treatment in this review are based on preclinical models, and their clinical efficacy remains uncertain.

Despite these advancements, several research gaps persist. Current studies predominantly focus on pathological, physiological, biomechanical, and imaging aspects of CEP degeneration, with limited exploration of the genetic and immunological factors involved. Additionally, while some international research has examined degenerative spinal diseases from a bacteriological perspective, more comprehensive investigations are needed. Most existing studies on both Western and Chinese medicine treatments targeting the CEP have been limited to *in vitro* experiments, with relatively few *in vivo* studies.

Future research should delve deeper into the genetic, immunological, and microbiological aspects of CEP degeneration. Collaboration between Western and Chinese medicine holds great potential to offer new insights into the pathogenesis and regenerative repair strategies for IDD. Advancing these interdisciplinary approaches will not only enhance our understanding of IDD but also facilitate the development of more effective therapeutic solutions.
